# Promotion of a synthetic degradation of activated STAT6 by PARP-1 inhibition: roles of poly(ADP-ribosyl)ation, calpains and autophagy

**DOI:** 10.1186/s12967-022-03715-x

**Published:** 2022-11-08

**Authors:** Jeffrey Wang, Mohamed A. Ghonim, Salome V. Ibba, Hanh H. Luu, Yucel Aydin, Peter A. Greer, A. Hamid Boulares

**Affiliations:** 1 School of Medicine, The Stanley Scott Cancer Center/Louisiana Cancer Research Center, Louisiana State University Health Sciences Center, New Orleans, LA USA; 2 Department of Microbiology and Immunology, Faculty of Pharmacy, Al-Azhar University, Cairo, Egypt; 3 Department of Pathology and Laboratory Sciences, Tulane University Health Science Center, New Orleans, USA; 4 Division of Cancer Biology and Genetics, Cancer Research Institute, Kingston, ON Canada

**Keywords:** Autophagy, Calpains, Poly(ADP-ribosyl)ation, Th2 inflammation, Olaparib, PARP-1, Protein degradation, Therapeutics

## Abstract

**Background:**

We reported that PARP-1 regulates genes whose products are crucial for asthma, in part, by controlling STAT6 integrity speculatively through a calpain-dependent mechanism. We wished to decipher the PARP-1/STAT6 relationship in the context of intracellular trafficking and promoter occupancy of the transcription factor on target genes, its integrity in the presence of calpains, and its connection to autophagy.

**Methods:**

This study was conducted using primary splenocytes or fibroblasts derived from wild-type or PARP-1^−/−^ mice and Jurkat T cells to mimic Th2 inflammation.

**Results:**

We show that the role for PARP-1 in expression of IL-4-induced genes (e.g. *gata-3*) in splenocytes did not involve effects on STAT6 phosphorylation or its subcellular trafficking, rather, it influenced its occupancy of *gata-3* proximal and distal promoters in the early stages of IL-4 stimulation. At later stages, PARP-1 was crucial for STAT6 integrity as its inhibition, pharmacologically or by gene knockout, compromised the fate of the transcription factor. Calpain-1 appeared to preferentially degrade JAK-phosphorylated-STAT6, which was blocked by calpastatin-mediated inhibition or by genetic knockout in mouse fibroblasts. The STAT6/PARP-1 relationship entailed physical interaction and modification by poly(ADP-ribosyl)ation independently of double-strand-DNA breaks. Poly(ADP-ribosyl)ation protected phosphorylated-STAT6 against calpain-1-mediated degradation. Additionally, our results show that STAT6 is a bonafide substrate for chaperone-mediated autophagy in a selective and calpain-dependent manner in the human Jurkat cell-line. The effects were partially blocked by IL-4 treatment and PARP-1 inhibition.

**Conclusions:**

The results demonstrate that poly(ADP-ribosyl)ation plays a critical role in protecting activated STAT6 during Th2 inflammation, which may be synthetically targeted for degradation by inhibiting PARP-1.

## Background

Our laboratory made major efforts to establish a role for poly(ADP-ribose)polymerase (PARP)-1 in asthma using several models of the disease [1–5]. Others confirmed our findings [6–9]. PARP-1 is a prominent member of the PARP protein family and is classically characterized as a DNA repair enzyme. We demonstrated that PARP is activated in lung and peripheral blood mononuclear cells (PBMCs) of asthmatics [10, 11]. The PARP-1 V762A polymorphism, which decreases enzymatic activity by 40% and is associated with reduced risk of asthma in humans [12] ultimately exemplifies the relationship between PARP-1 and asthma. We reported that PARP inhibition by next-generation drugs such as olaparib or gene KO blocks established asthma-like traits in mice chronically exposed to ovalbumin (OVA) or house dust mite extracts (HDM) [1–5]. We demonstrated that PARP-1 regulates the expression of many genes whose products are crucial in asthma by controlling NF-κB nuclear trafficking and the fate of STAT6 following IL-4 or allergen exposure [3, 5, 6, 13–15]. The effect of PARP-1 inhibition on STAT6 integrity prevents the expression of GATA-3 [10], the master regulator of the *IL4/IL5/IL13* cytokine locus [16]. PARP-1 inhibition appears to destabilize STAT6 on IL-4 or allergen exposure potentially through a calpain-dependent mechanism as a partially specific calpain inhibitor (ALLN) reversed this process [5]. Although the kinetics and dynamics of STAT proteins have received great attention, little is known about the mechanism by which STAT6 is regulated upon activation. The majority of published reports show no degradation of STAT6 on activation by IL-4, IL-13, or allergens. Very few reports showed STAT6 degradation by calpains [17, 18] or other proteases [18–20] including a non-calpain-generated truncation product found only in mast cells [18, 21, 22]. A critical aspect of the aforementioned STAT6 degradation is its association with pro-inflammatory outcomes. Conversely, however, PARP-1 inhibition-related STAT6 degradation is associated with an anti-inflammatory outcome [5].

Calpains are neutral cysteine proteases that are activated by calcium [23] of which calpain (CAPN)-1/2 are the most extensively-investigated [24, 25]. Calpains are composed of a catalytic subunit that forms a heterodimer with a regulatory calpain small subunit 1 (CAPNS1) [26]. Proteolytic activity of calpains can be controlled by calpastatin, a stable CAPN1/2-specific peptide substrate inhibitor that is expressed endogenously [27]. There are no known target consensus sequences for cleavage by calpains, with few exceptions via the PEST sequence [28]. Even though calpain activation leads to a more severe manifestation of asthma in the murine model [29], inhibition of calpain in PBMCs leads to a suppression in Th1/Th17 but an increase in Th2 cytokine production [30, 31]. Moreover, calpains appear to play an important role in autophagy and both processes are known to be induced by allergens [32]. Autophagy is a complex process and encompasses several types that include macroautophagy, microautophagy, and chaperone-mediated autophagy (CMA) [33]. Macroautophagy is responsible for the degradation of the majority of the proteins substrates. Microautophagy plays a smaller role and guides its substrates to the lysosome. CMA targets proteins with the consensus sequence KFERQ-like motif for HSP70-mediated transport into the lysosome for degradation [34, 35].

In this study, we examined the nature of PARP-1/STAT6 relationship in terms of both physical interaction and poly(ADP-ribosyl)ation (PARylation). Given that our conclusion on the calpain/STAT6 relationship was based on the use of a non-specific inhibitor, we wished to determine whether PARP-1 inhibition-associated degradation of the transcription factor is, indeed, calpain-mediated. Because of the dichotomy associated with the kinetics and dynamics of STAT6 activation and PARP inhibition-associated degradation of the transcription factor, we examined whether PARP-1 regulates STAT6 through nuclear trafficking upon IL-4 stimulation. Finally, given the connection between calpains, autophagy, and Th2 inflammation, we sought to examine the fate of STAT6 during autophagy; and if the fate were altered, we would determine whether PARP-1-mediated modification of IL-4-activated STAT6 interferes with calpain-mediated degradation.

## Materials and methods

### Animals

C57BL/6 J Wild type and PARP-1^−/−^ mice (5–8 weeks old) were bred and maintained in a specific-pathogen free facility at Louisiana State University Health Sciences Center of New Orleans with unlimited access to sterilized chow and water. All experimental protocols and procedures were approved by the IACUC.

### Cell culture, splenocyte isolation and treatment, subcellular fractionation, immunoprecipitation (IP), chromatin immunoprecipitation (ChIP) and immunoblot analysis

Cells and treatments were conducted as described in [5]. Whole cell extracts or cell fractions were prepared using standard protocols. The antibodies used in this study are as follows: STAT6 (BioLegend); STAT2, STAT1, STAT4, β-Actin or GAPDH (Santa Cruz Biotechnology); STAT3, GATA-3, or α-Tubulin (Cell Signaling Technology); p-STAT6 (Y641) (Life Technologies).

ChIP was performed using the EZ-Magna ChIP^™^ G–Chromatin Immunoprecipitation Kit following manufacturer’s protocols. Isolated DNA was assessed by qPCR using specific primers for the *gata-3* proximal or  distal promoter. The primers are as follows: *gata-3* proximal promoter forward: 5ʹ-ATGCATCGCGTTGTCACTAA-3ʹ; *gata-3* proximal promoter reverse: 5ʹ-CCAAACCTCTCCAGAACGAA-3ʹ; *gata-3* distal promoter forward: 5ʹ-TGCCTATGATAATGGCCCATTC-3ʹ; *gata-3* distal promoter reverse: 5ʹ-CTGCTCCTGGTGCCTACAAAG-3ʹ.

### Cell-free phosphorylation, PARylation, and calpain enzymatic reaction

Recombinant human STAT6 (400 ng) (Sino Biological) was incubated with 100 ng of recombinant human JAK3 (Active Motif) in kinase buffer and then incubated with 1.5 mM ATP (Affymetrix) to start the reaction for 30 min at 37 °C. For the assessment of PARylation, reaction buffer and 100 ng of recombinant human PARP-1 (Active Motif) were added to the reaction mixture, with 2 mM NAD^+^ (Abcam) and then incubated for 30 min at 37 °C. For the calpain enzymatic reaction, 34 ng of recombinant human calpain-1 (BioVision) were added to calpain buffer and incubated at 37 °C. The reaction samples either underwent Ni–NTA pulldown or were terminated with the addition of SDS sample buffer and heating at 95 °C for 5 min. The terminated reactions were loaded onto an SDS-PAGE gel for subsequent immunoblot analysis.

### Autophagy induction and treatments

Cells were exposed to autophagy media for the indicated time periods in the presence or absence of indicated amount of amino acid supplementation. In some experiments, the cells were also incubated in the presence or absence of recombinant human IL-4, olaparib, calpastatin, 3-methyladenine (3- MA), Torin 1, Hydroxychloroquine (HQC) in combination or individually as described below. Protein extracts were then subjected to immunoblot analysis with the appropriate antibodies.

### Data analysis

All data are expressed as the mean ± SEM of values from triplicates samples for qPCR. Experiments were conducted at least three times. PRISM software (GraphPad) was used to analyze the differences between experimental groups by one-way ANOVA followed by Tukey method.

## Results

### PARP-1 influences GATA-3 expression in IL-4-stimulated splenocytes without affecting STAT6 phosphorylation or its subcellular trafficking but by influencing its occupancy of the *gata-3* promoter

**Fig. 1:**
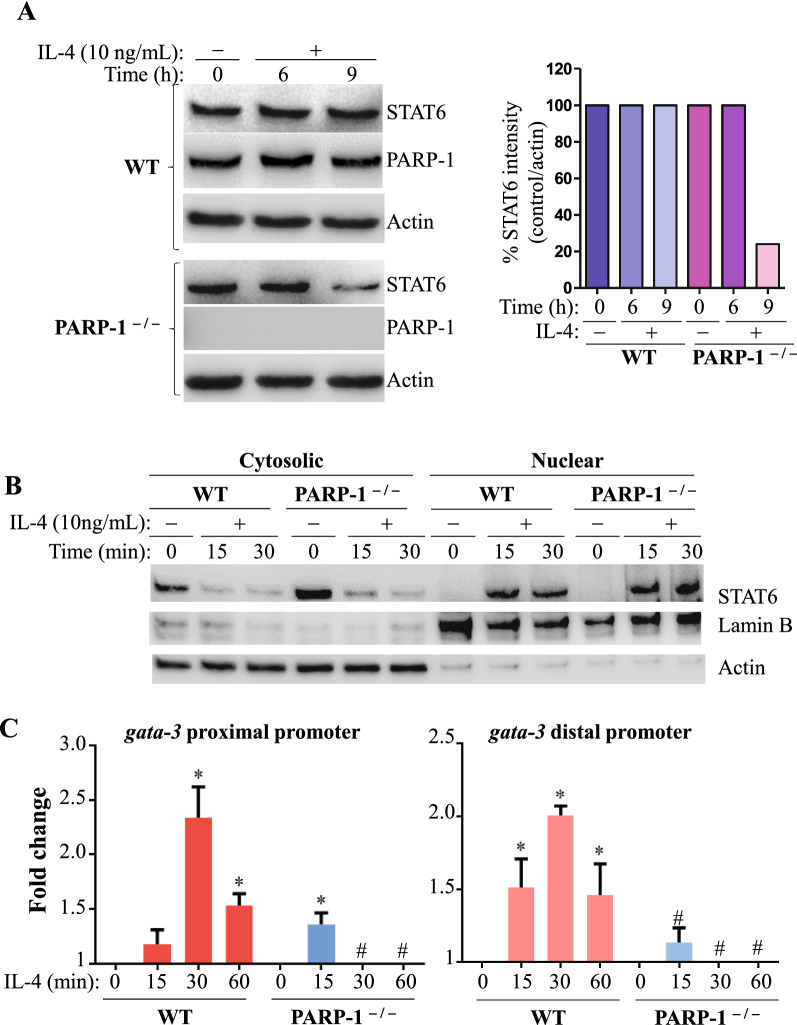
*PARP-1 influences STAT6 protein level and regulates its occupancy on the gata-3 promoter following IL-4 stimulation.* **A** WT and PARP-1^−/−^ splenocytes isolated from C57BL/6 J mice were stimulated with IL-4 for the indicated time points. Protein lysate were analyzed for STAT6, pSTAT6 (Y641), PARP-1 and Actin. The bands of STAT6 were quantified and expressed as percent change compared with their respective untreated control/actin (right panel). **B** IL-4-treated cells and relative controls undergo subcellular fractionation and then protein extracts were analyzed for STAT6, Lamin B and Actin. **C** WT and PARP-1 ^−/−^ splenocytes isolated from C57BL/6 J mice were stimulated with IL-4 for the indicated intervals of time. ChIP for STAT6 was performed and immunoprecipitated DNA was analyzed using qPCR targeting the proximal or distal promoter of *gata-3.* Data are representative of at least three independent experiments. *, # Significant difference from respective controls or experimental sample, respectively (*p* < 0.05)

Figure 1A shows that PARP-1 gene deletion does not affect IL-4-induced STAT6 phosphorylation at Y641 at least for the first 6 h or its translocation from the cytosol to the nucleus (Fig. 1B). Because STAT6 phosphorylation and subsequent subcellular trafficking were not affected by PARP-1 gene knockout, this implied that the signaling pathway upstream of this process was not appreciably impacted by a lack of the enzyme. This prompted a search for further downstream elements by focusing on the role of PARP-1 in STAT6 occupancy of promoters of target genes, namely, *gata-3*. Stimulation of WT splenocytes isolated from C57BL/6 J mice with IL-4 induced STAT6 binding to the proximal or the distal part of the *gata-3* promoter after 30 min, as assessed by ChIP assay with antibodies to STAT6 and DNA amplified by primers specific to the *gata-3* promoter*.* However, in PARP-1^−/−^ splenocytes, *gata-3* promoter occupancy by STAT6 after IL-4 treatment was markedly reduced (Fig. 1C), corroborating the diminished GATA-3 expression observed in these conditions [5]. Interestingly, there was either no significant difference between the IL-4-treated PARP-1^−/−^ splenocytes and the untreated WT splenocytes, or the signal was below the background level (using normal IgG). These results suggest a requirement of PARP-1 for the persistent binding of STAT6 to the *gata-3* promoter after its activation and nuclear translocation.

### STAT6 interacts with and is PARylated by PARP-1 in a cell-free system and in IL-4-treated splenocytes and such PARylation protects the transcription factor against degradation by calpain-1

**Fig. 2:**
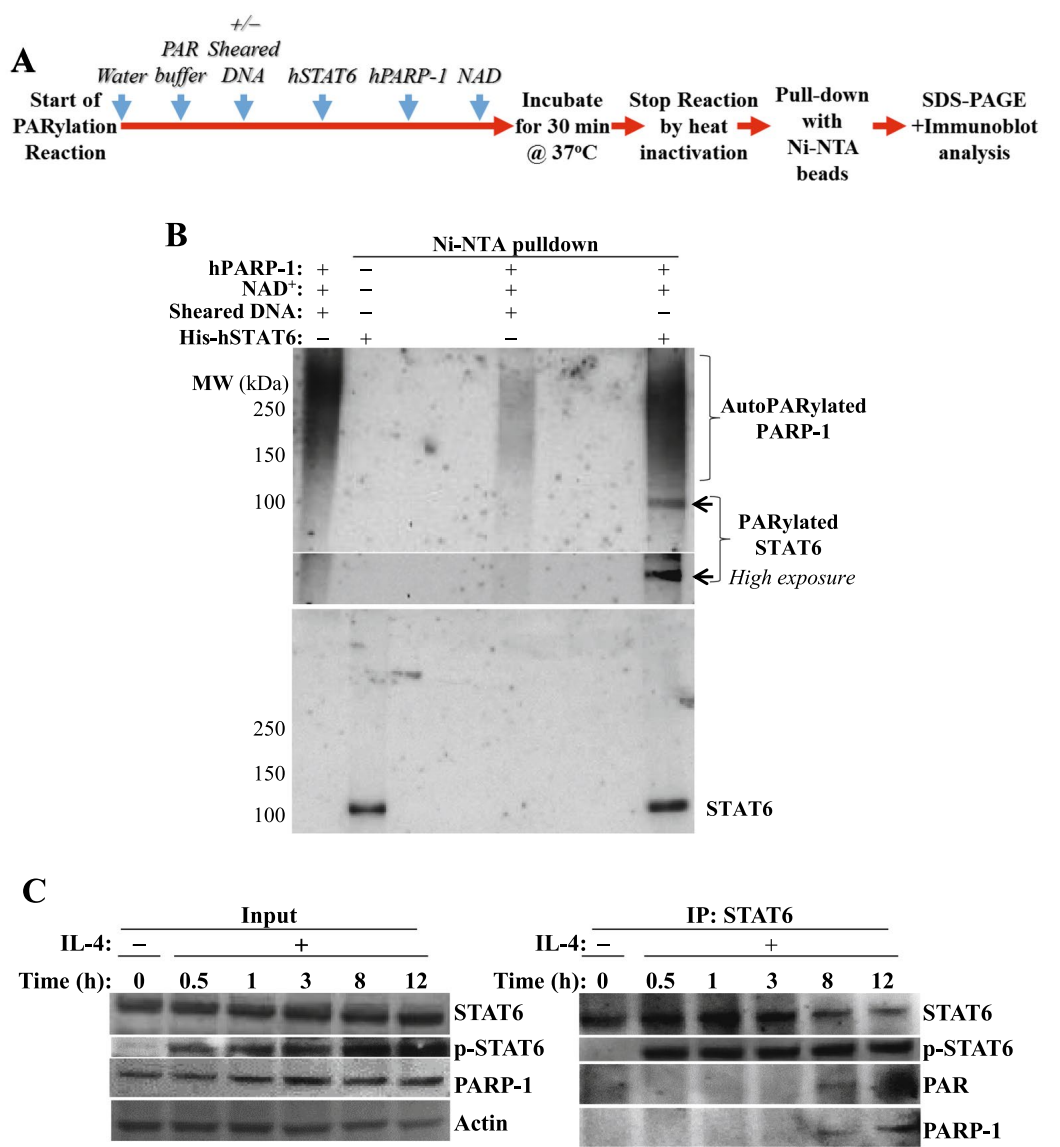
*STAT6 interacts with and is PARylated by PARP-1 in a cell-free system with recombinant proteins and in IL-4 stimulated mouse splenocytes.* **A** Workflow of the cell-free PARylation reaction. **B** Ni–NTA pull down for hSTAT6 was performed and samples were examined with antibodies against PAR, or STAT6. **C** IL-4-treated splenocytes and controls were collected at the indicated time points. Protein extracts were subjected to immunoprecipitation (IP) with antibodies to STAT6. Subsequently, an immunoblot analysis of the IP material or input proteins was performed using antibodies against STAT6, p-STAT6 (Y641), PAR, PARP-1, or Actin. Data are representative of at least 3 independent experiments

Physical interaction and/or PARylation may be necessary for PARP-1 to regulate STAT6 function and integrity. To this end, a cell-free recombinant PARylation reaction system was employed, along with Ni–NTA pulldown to enrich polyHis-tagged STAT6 as outlined in Fig. 2A. The conventional PARylation reaction requires PARP-1, sheared DNA for activation as a source of double-stand DNA breaks (DSBs), and NAD^+^ as the coenzyme for PARP-1 to form long chains of PAR polymers. The resultant product separated by immunoblotting and probed with antibody against PAR typically displays smear-like bands especially when no substrate is added (Fig. 2B). The PARylation reaction revealed a distinct band, which was located at the same molecular weight as STAT6, suggesting PARylation. PARP-1 was also pulled down along with STAT6 in the same reaction, clearly indicating a physical interaction between the two proteins. Surprisingly, this interaction and PARylation did not require DSBs suggesting that PARP-1 may modify STAT6 independently of DNA damage. To eliminate the possibility that PARP-1 was activated in the PARylation reaction non-specifically by potential contaminant DNA, the reaction was incubated with recombinant DNase1 prior to addition of NAD^+^; the addition of the endonuclease did not change the PARylation pattern (data not shown). Figure 2C shows that although PARylation of STAT6 and interaction with PARP-1 did occur, this took place much later after IL-4 stimulation (around 8 to 12 h). Interestingly, this corresponds to the time when STAT6 is beginning to be degraded in PARP-1^−/−^ cells [5], emphasizing the temporal nature of this phenomenon.

### Phosphorylated STAT6 is selectively degraded by Calpain-1 and is protected by the endogenous inhibitor, calpastatin, or PARP-1-mediated PARylation.

**Fig. 3:**
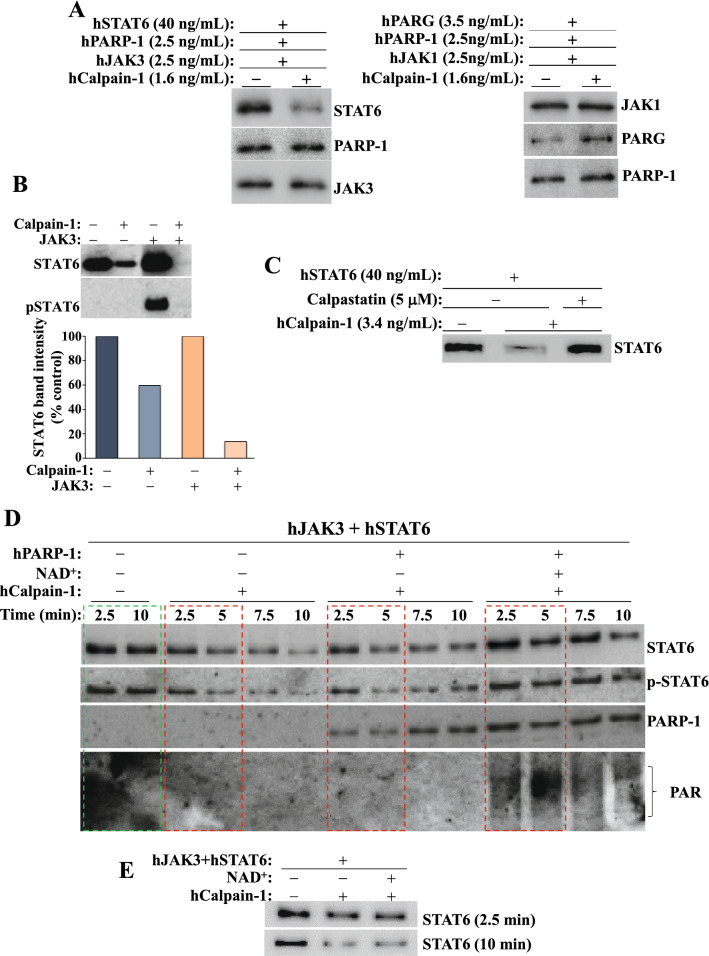
*STAT6 is selectively degraded by Calpain-1 but its PARylation protects it against this process*. **A** Recombinant poly-His-STAT6 was phosphorylated in vitro by active JAK3 or left unphosphorylated prior to incubation with calpain-1 for 10 min. The reaction was stopped with sample buffer and analyzed by immunoblot analysis. The bands were quantified and expressed as percent change compared with their respective untreated control (bottom panel). **B** Calpain-1 was incubated with STAT6, JAK3, JAK1, PARP-1, or PARG for 40 min. Immunoblot analyses were performed with antibodies against STAT6, PARP-1, JAK1, JAK3, or PARG. **C** Calpastatin was added to the cell free reaction mix containing STAT6 and Calpain-1. Samples were then subjected to immunoblot analysis with antibodies to the respective proteins. **D** JAK3-mediated STAT6 phosphorylation, PARylation, and calpain enzymatic reactions were performed in this order. Immediately after the phosphorylation the reaction was divided in 4 parts.Then, immunoblot analysis was carried out with antibodies against STAT6, p-STAT6 (Y641), PARP-1, or PAR. **E** Recombinant STAT6 was incubated with JAK3 in the presence of calpain-1 with or without NAD^+^. STAT6 degradation was assessed by immunoblot analysis

Figure 3A shows that although STAT6 is degraded by calpain-1, phosphorylation by JAK3 renders it more susceptible to degradation by the protease. To test substrate selectivity of calpain-1, the enzyme was incubated with the different components of the IL-4/JAK/STAT pathway along with PARP-1 and the PAR glycohydrolase (PARG). Remarkably, STAT6 was the only protein degraded after a 40 min incubation with the protease even upon using large amounts of the protein (Fig. 3B), indicating that STAT6 may be a selective substrate for the protease, and that it may possess inherent motifs that render it susceptible to degradation by the protease. Addition of calpastatin, which prevents calpain self-activation upon exposure to calcium [36], blocked calpain-1-mediated degradation of STAT6 (Fig. 3C). Interestingly, an examination of the STAT6 protein for the presence of potential PEST-like motifs for selective degradation by calpains [37] revealed that no such sequences exist on the protein.

The cell-free reaction was started with a phosphorylation of STAT6 by active JAK3. Compared with the control reactions, which do not include the protease (Fig. 3D, green box), addition of calpain-1 showed a time-dependent degradation of STAT6 (red boxes). The phosphorylated form p-STAT6 was equally degraded by the protease. Interestingly, addition of PARP-1 to the reaction mix did not alter the extent of STAT6 degradation by calpain-1. However, supplementation of the reaction with NAD^+^, the coenzyme of PARP-1, provided excellent protection against calpain-1-mediated STAT6 degradation. Co-incubating STAT6 and JAK3 in the presence of calpain-1 did not protect the transcription factor from degradation when NAD^+^ was excluded from the reaction. This emphasizes the requirement of PARP-1 activity and subsequent PARylation of STAT6 for the observed protective effects. This notion is supported by the observation that the addition of NAD^+^ alone without PARP-1 did not protect STAT6 against calpain-1 (Fig. 3E).

### STAT6 is degraded by AA starvation-induced CMA in a selective and calpains-dependent manner

**Fig. 4:**
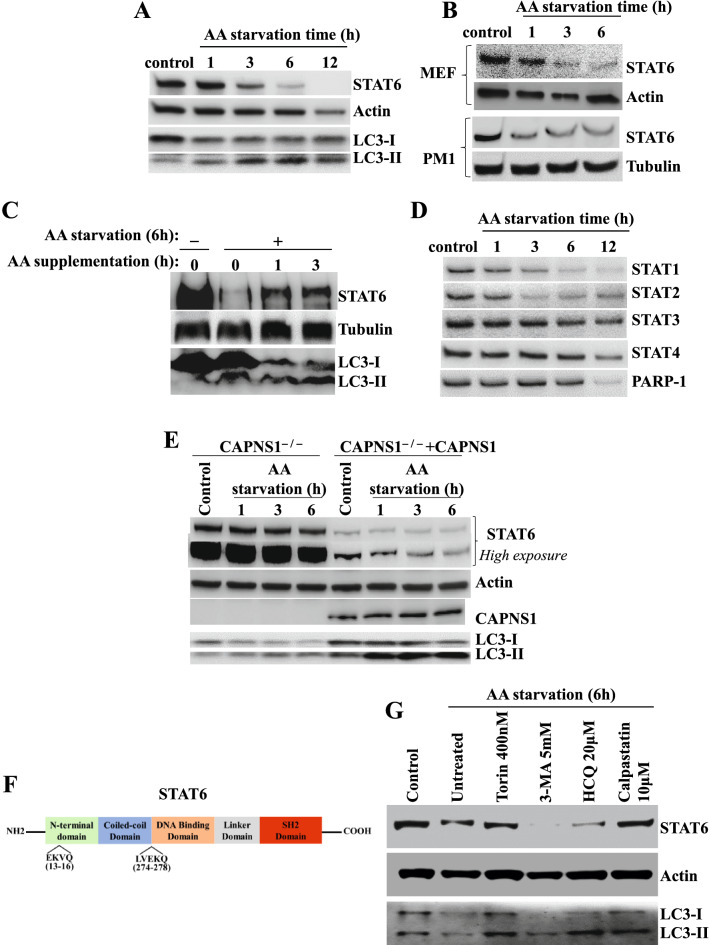
*STAT6 is degraded by AA starvation-induced autophagy*. **A**–**B** Jurkat, MEF and PM1 cells were exposed to AA starvation media for the indicated time and then processed for immunoblot analysis. Antibodies against STAT6, LC3, actin, or tubulin were used.  **C** Jurkat cells were cultured for 6 h in starvation media and then supplemented with AA for the indicate times. Protein extracts were subjected to immunoblot analysis with antibodies to STAT6, tubulin, or LC3. **D** The blot from (**A**) was re-probed with antibodies to STAT1, STAT2, STAT3, STAT4 or PARP-1. **E** CAPNS1^−/−^ cells expressing human *CAPNS1* or empty vector were subjected to AA starvation for the indicated times. Protein extracts were then subjected to immunoblot analysis with antibodies to STAT6, CAPNS1, LC3 or actin. **F** Putative CMA-targetable motifs on STAT6. **G** Jurkat cells subjected to AA starvation for 6 h were concomitantly treated with CMA inducers or inhibitors or calpastatin. Levels of STAT6 and autophagy status were assessed by immunoblot analysis of protein extracts with antibodies to STAT6, LC3 or actin

Allergen challenge in mice induces autophagy and calpain activation [38]. Calpain is required for autophagosome formation in macroautophagy [39], and PARP-1 inhibition may induce autophagy [40, 41]. We, thus, examined the dynamics of STAT6 upon an induction of autophagy by amino acid (AA) starvation. Figure 4A shows that AA starvation promoted a rather slow but time-dependent decrease in STAT6 protein levels in Jurkat T cells, which coincided with induction of autophagy signaled by the turnover of LC3-I to LC3-II, the lipidated form of LC3. Although STAT6 degradation was first observed at 3 h post-starvation, maximum degradation occurred only after 12 h of AA starvation. This phenomenon was not restricted to Jurkat T cells as it was also observed in other cell types including the CD4^+^ T cell line, PM1 cell and MEFs (Fig. 4B) as well as the lung epithelial cell line, A549 (data not shown). Figure 4C shows that the recovery of STAT6 upon induction of autophagy was relatively fast as it was detectable as early as 1 h after AA supplementation, which may suggest that STAT6 mRNA levels were not affected. Similar to STAT6, degradation of STAT1, STAT2, and STAT3 was observed during autophagy although STAT3 was degraded in a much slower pace than the other STAT proteins (Fig. 4D). Conversely, however, STAT4 remained unaffected after 6 h of AA starvation.

To reinforce the notion that calpain plays a role in regulating STAT6 levels, we used *CAPNS1*^*−/−*^ MEFs transduced by a lentiviral vector encoding human *CAPNS1* or empty vector. Remarkably, STAT6 was drastically elevated in *CAPNS1*^*−/−*^ cells compared to cells that expressed CAPNS1 (Fig. 4E) clearly demonstrating that STAT6 levels are regulated by calpains regardless of autophagy. Induction of autophagy in *CAPNS1*^*−/−*^ MEFs did not cause an obvious degradation of STAT6 while the rescue of CAPNS1 in these cells promoted a time-dependent degradation of the transcription factor.

AA starvation is known to be a strong promoter of CMA [42]. Our results shown in Fig. 1A indicate, perhaps, that the early degradation of STAT6 may be attributed to macroautophagy, the later (~ 12 h of AA starvation) degradation may be CMA-mediated. Interestingly, an examination of STAT6 protein sequence revealed the existence of two putative CMA motifs [42] (Fig. 4F) between residues 13–16 (EKVQ) and 274–278 (LVEKQ). To investigate the influence of autophagy on STAT6 integrity, we examined the fate of the transcription factor in condition conducive to induction of autophagy and used either autophagy inducers or blockers. It is interesting that treatment of cells with Torin1, a bonafide inducer of macroautophagy [43], exerted no additional effect on STAT6 degradation upon AA starvation, while treatment with established inhibitors of macroautophagy, 3-MA or HCQ, did not block degradation of the transcription factor (Fig. 4G). The dynamics of LC3B I/II in the different experimental conditions provide evidence of the expected effects of the tested drugs in the in vitro system. Conversely and interestingly, however, the calpain inhibitor, calpastatin, markedly blocked the degradation of STAT6 without blocking autophagy as indicated by the higher levels of lapidated LC3-II. These results strongly suggest that CMA may be the predominant mediator of STAT6 degradation during autophagy. It is important to note that this is the first study reporting STAT6 as a new substrate for CMA.

### IL-4 treatment partially protects STAT6 from CMA-mediated degradation but PARP-1 inhibition abrogates such protection.

**Fig. 5:**
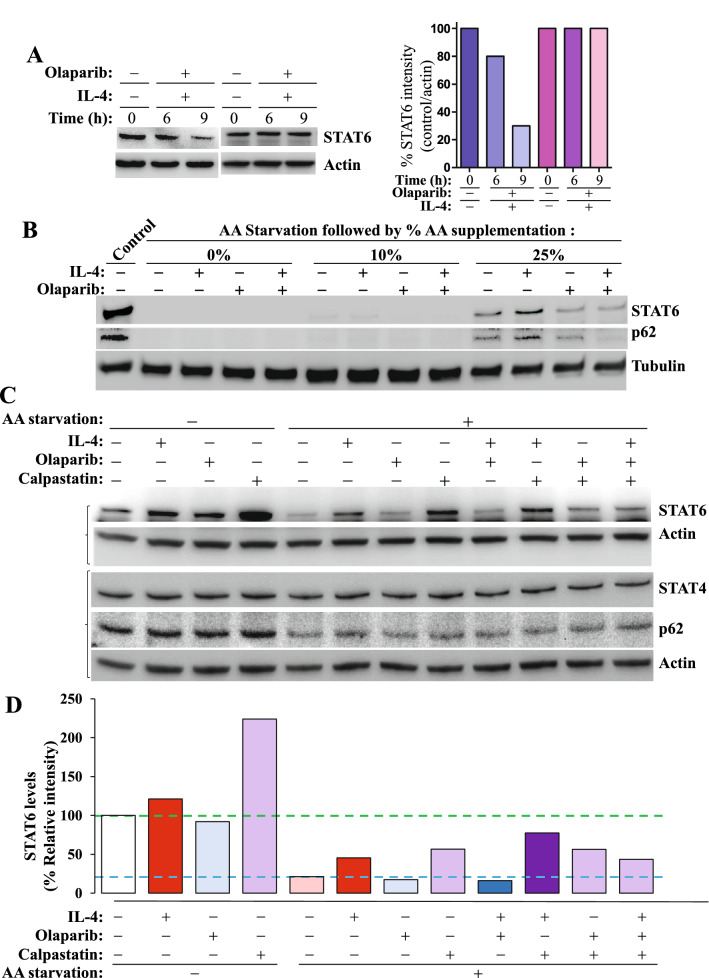
*IL-4 treatment protects STAT6 from autophagy-induced degradation and PARP-1 inhibition abrogates such protection without affecting autophagy*. **A** Splenocytes isolated from WT C57BL/6 J mice were stimulated with IL-4 in the absence or presence of olaparib for the indicated time points. Protein lysates were subjected to immunoblot analysis with antibodies to STAT6 or Actin. **B** Jurkat cells were exposed to AA starvation media then supplemented with different percentages of AAs in the presence or absence of IL-4 or the PARP inhibitor, olaparib, for 12 h. Protein extracts were then subjected to immunoblot analysis with antibodies to STAT6, p62 or Tubulin. The brackets on the left indicate that the two sets of panels were of same samples but run on two different gels. **C** Jurkat were cultured in starvation media then supplemented with 25% AAs and treated for 12 h with IL-4, olaparib, calpastatin or combinations of the different agents. Not starved cells were used as control. Protein extracts were then subjected to immunoblot analyses with antibodies to STAT6, STAT4, p62 or actin. **D** The intensity of the STAT6 bands showed on (**B**) was quantified using ImageJ-Fiji and results were normalized to respective Actin band intensity

Similarly to the effect of PARP-1 gene knockout, inhibition of PARP with its clinically used inhibitor, olaparib, also promoted a decrease in the levels of STAT6 upon stimulation with IL-4 (Fig. 5A). The addition of IL-4 with or without olaparib did not rescue STAT6 degradation in strong autophagy conditions (Fig. 5B). To reduce the extent of autophagy, AA was supplemented to the autophagy medium. A 10% AA supplementation failed to generate any effect of IL-4 treatment on STAT6 levels. However, with a 25% AA supplementation, STAT6 began to appear in unstimulated cells, which was enhanced upon treatment with IL-4. Treatment with olaparib reduced the basal levels of the transcription factor as well as those promoted by IL-4 treatment (Fig. 5B). The dynamics of STAT6 in the aforementioned conditions mirrored rather small changes in sequestosome-1 (p62) levels (Fig. 5C). As expected, inhibition of calpain with calpastatin promoted an accumulation of STAT6 in the absence of any stimulus and blocked its autophagy-induced degradation (Fig. 5C–D); IL-4 did not promote additional accumulation of STAT6. Surprisingly, treatment with olaparib appeared to impair calpastatin-mediated rescue of STAT6. Figure 5C also shows that autophagy, as assessed by p62 levels, remained relatively unchanged and whatever minor changes occurred, they did not explain the dynamics of STAT6. As expected, STAT4 levels were not affected by any of the conditions or treatments clearly suggesting its resistance to autophagy.

## Discussion

**Fig. 6:**
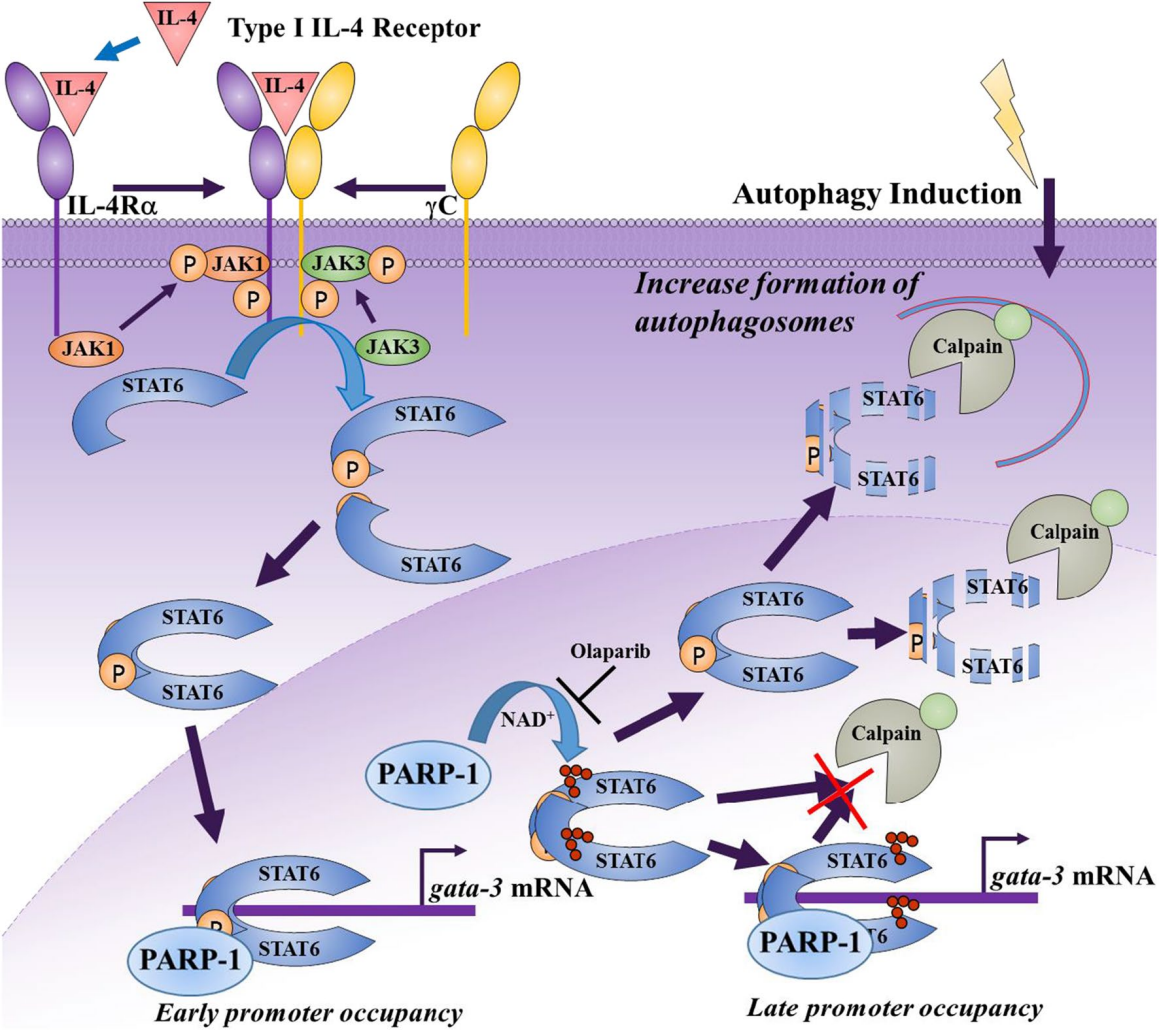
*Synthetic degradation of IL-4-activated STAT6 upon PARP inhibition and its association with calpains and autophagy.* IL-4 binds to its receptor (upon the dimerization of the IL-R4α and γC subunits) leading to the recruitment and subsequent activation of JAK1/3 kinases, which culminates in the phosphorylation of STAT6. Phosphorylated STAT6 monomers then dimerize and translocate to the nucleus where they occupy the *gata-3* gene promoter. During the early stages after IL-4 stimulation, PARP-1 expression is required for a persistent occupancy of the *gata-3* promoter by the STAT6 dimer. Whether PARylation is required here is not clear. At a later stage, phosphorylated STAT6 is PARylated, which protects it from calpain-mediated degradation. Inhibition of PARylation by PARP inhibitors renders STAT6 susceptible to degradation. Autophagy also promotes degradation of STAT6 and is protected by IL-4 stimulation and subsequent PARylation. CMA appears to be the major mechanism by which STAT6 is degraded during autophagy. The increased susceptibility of phosphorylated STAT6 for degradation by calpains upon PARP inhibition may be considered as artificial or synthetic, hence our proposal of naming this process “*synthetic protein degradation*”

The present study demonstrates, for the first time, that STAT6 interacts with and is PARylated by PARP-1. PARylated STAT6 only accumulates to detectable levels much later after IL-4 stimulation. This may mean that the extent of modification on STAT6 is very limited and undetectable by the utilized approach (Fig. 6). It is unclear whether PARylation of STAT6 is involved in the regulation of its occupancy of the *gata-3* promoter. The relationship between STAT6 and PARP-1 does not seem to require DNA damage, which enlarges the number of situations where the enzyme functions independently of its role in DNA repair. This is consistent with our recent report showing that MDSC function can be affected by PARP inhibition with a sub-IC50 concentration of the drug, olaparib, without promoting DNA damage or the STING pathway [44]. The results of this study also unravels an interesting connection between STAT6 and its activation by IL-4, autophagy, and PARP-1. It is noteworthy that this is the first study reporting that STAT6 may be a novel substrate to CMA with putative targeting motifs.

The connection between STAT6 phosphorylation, PARylation and susceptibility to degradation by calpains upon PARP inhibition is of the utmost importance. Because PARP-1 inhibition is not often considered as natural given that few cellular factors are known to directly inhibit PARP-1 [45], the increased susceptibility of phosphorylated STAT6 for degradation by calpains upon PARP inhibition may be considered as artificial or synthetic (Fig. 6). Therefore, this sensitization may present itself as a unique opportunity that could be employed to promote a reduction in IL-4-mediated signaling. We consider this process as a completely novel phenomenon, which we now term “*synthetic protein degradation*” that is related to activated STAT6. This concept is different from what is known as “Targeted protein degradation” through the proteolysis targeting chimeric (PROTAC) technology via the ubiquitin–proteasome system [46]. Synthetic protein degradation involves a completely different mechanism and uses calpains as mediators of degradation. While our concept targets activated STAT6, PROTAC, as designed for STAT3 in cancer [47, 48], for instance, does not differentiate between active *vs*. inactive transcription factors. With the critical function of STING/TBK1-dependent but IL-4Rα/JAK-independent STAT6 activation in antiviral innate immunity, targeting STAT6 via PROTAC may be problematic. Given the finding that phosphorylated STAT6 is the primary target for degradation upon PARP-1 inhibition, this represents a unique opportunity to target the transcription factor for degradation during asthma where the IL-4/IL-13/STAT6 pathway is upregulated especially in uncontrolled/severe disease [49]. According to our results, calpains may also influence the integrity of STAT6 in physiological conditions (Fig. 4E). However, we surmise that the degradation may be too low to be obvious, which may explain that such observation was never reported.

According to our results, STAT6, when in the activated state, can be targeted for degradation by calpains by preventing its modification by PARP-1. This constitutes a paradigm shift concept where activated STAT6, not its unstimulated form, is the ideal target for degradation. This stems from the fact that asthma is associated with enhanced IL-4-associated STAT6 phosphorylation and subsequent production of Th2 cytokines [49]. Targeting activated STAT6 for degradation may not be unique to asthma; rather, it can be applied to cancer as well. Indeed, in tumor-associated macrophages, targeting STAT6 was shown to reduce tumor growth and metastatic niche formation in breast cancer [50].

PARP-1 regulates the subcellular trafficking of transcription factors through its PARylation [15], and GATA-3 protein expression is reduced in IL-4-stimulated PARP-1^−/−^ splenocytes around the same time as STAT6 degradation [5], indicating an effect on signal transduction or transcription. Together with the fact that degradation of STAT6 by calpain occurs in the cytosol [51, 52], this implies that STAT6 may be regulated through the same mechanism. Interestingly, however, subcellular trafficking of STAT6 does not appear to be dependent on the presence of PARP-1 in splenocytes, which suggests a different mechanism at play. Our results indicate that *gata-3* promoter occupancy by STAT6 is affected by PARP-1, lending to the probable dual function of PARP-1 in regulating STAT6 via transcriptional activation and protein stability. The mechanism behind the effect on transcription may be similar to PARylation of HDACs by PARP-14, which releases them to allow access to the promoter [53, 54].

Very few reports dealt with the connection between STAT6 and autophagy. In diabetic kidney disease where autophagy is dysregulated, STAT6 is upregulated [55]. Recently, bixin, a carotenoid natural product, was shown to protect against kidney interstitial fibrosis by promoting STAT6 degradation through ubiquitination-associated autophagy [56]. Interestingly, when autophagy is induced by LPS, the transcription factor remains intact [57]. No report has shown a degradation of STAT6 during allergic asthma although the condition, especially when severe, induces autophagy in many cell types including B cells [58] and neutrophils [59]. Our results provide strong evidence that STAT6 is specifically degraded by CMA as it harbors specific motifs. More importantly, STAT6 degradation is prevented by IL-4 stimulation, PARP-1, and calpastatin.

## Conclusion

We believe that the results of our study not only provide a stronger support for the role of PARP-1 in asthma, but they also introduce synthetic STAT6 degradation as a novel concept in the field of asthma to block lung inflammation and remodeling, as well as provide novel insights into the regulation of activated STAT6. Finally, the knowledge to be gained upon additional studies will be applicable to many transcription factors or enzymes that can be targeted for degradation (or stability) to block or dampen the negative effects of associated diseases.

## Data Availability

Not applicable.

## Abbreviations

CAPNS1: Regulatory calpain small subunit 1
CMA: Chaperone-mediated autophagy
Gata-3: GATA binding protein 3
PAR: Poly(ADP-ribose)
PARP-1: PAR polymerase
PARG: PAR glycohydrolase
PARylated: Poly(ADP-ribosyl)ated
PARylation: Poly(ADP-ribosyl)ation
STAT: Signal transducer and activator of transcription
